# Preparation of Crystalline LaFeO_3_ Nanoparticles at Low Calcination Temperature: Precursor and Synthesis Parameter Effects

**DOI:** 10.3390/ma14195534

**Published:** 2021-09-24

**Authors:** Wen Jiang, Liwei Cheng, Jianghui Gao, Shiyu Zhang, Hao Wang, Zhihao Jin, Zhongfeng Tang, Cheng Peng

**Affiliations:** 1 College of Mechanical and Power Engineering, Shenyang University of Chemical Technology, Shenyang 110142, China; jiangwen@sinap.ac.cn; 2 Key Laboratory of Interfacial Physics and Technology, Shanghai Institute of Applied Physics, Chinese Academy of Sciences, Shanghai 201800, China; chengliwei@sinap.ac.cn (L.C.); gaojianghui@sinap.ac.cn (J.G.); zhangshiyu@sinap.ac.cn (S.Z.); wanghao@sinap.ac.cn (H.W.); 3 University of Chinese Academy of Sciences, No. 19(A) Yuquan Road, Shijingshan District, Beijing 100049, China

**Keywords:** crystalline LaFeO_3_ nanoparticles, coprecipitation, calcination, FeO_6_ octahedra, perovskite

## Abstract

Substantial effort has been devoted to fabricating nanocrystalline lanthanum ferrite (LaFeO_3_), and calcination is the crucial process of crystallization in both high-temperature strategies and wet chemical methods. Lowering the calcination temperature gives the ability to resist the growth and agglomeration of nanoparticles, therefore contributing to preserve their unique nanostructures and properties. In this work, we prepared crystalline LaFeO_3_ nanoparticles with a calcination process at 500 °C, lower than the calcination temperature required in most wet chemistry methods. Correspondingly, the experimental conditions, including stoichiometric ratios, pH values, precipitants, complexant regent, and the calcination temperatures, were investigated. We found that the crystalline LaFeO_3_ was formed with crystalline NaFeO_2_ after calcination at 500 °C. Furthermore, the structure of FeO_6_ octahedra that formed in coprecipitation was associated with the process of crystallization, which was predominantly determined by calcination temperature. Moreover, an illusion of pure-phase LaFeO_3_ was observed when investigated by X-ray diffraction spectroscopy, which involves amorphous sodium ferrite or potassium ferrite, respectively. These findings can help prepare nanostructured perovskite oxides at low calcination temperatures.

## 1. Introduction

Lanthanum ferrite (LaFeO_3_, LFO) is a prominent ABO_3_-type perovskite oxide that has great potential applications in advanced technologies such as solid-oxide fuel cells and molten salt batteries owing to the unique electrochemical activities and thermal properties. Nowadays, a large number of synthetic methods to fabricate LFO-based perovskite oxides have been developed, including high-temperature strategies (i.e., solid-state reaction [1,2] and molten salt synthesis [3,4,5]) and wet chemical methods such as electrochemical deposition [6,7], electrospinning [8], microwave plasma [9], sol–gel [10,11,12,13,14,15,16], combustion [17], hydrothermal [18,19,20], and precipitation [21,22]. High-temperature strategies have been widely utilized for the fabrication of bulk LFO-based oxides that often require a calcination process at high temperatures (above 800 °C). Wet chemical methods are extensively utilized to fabricate LFO-based perovskite nanostructures that usually involve a sol–gel or coprecipitation process for precursor preparation and a calcination process for crystallization. Lowering the calcination temperature is vital for LFO-based perovskite nanostructures to avert morphology evolution and recrystallization [23,24,25].

On the other hand, calcination temperature is particularly associated with the crystallinity of LFO nanostructures. Lowering the calcination temperature is not only advantageous to avoid the particle growth and agglomeration during high-temperature processes, but also advantageous to decrease the energy consumption, as well as carbon dioxide emissions. For wet chemical methods, a calcination temperature above 600 °C is essential to prepare crystalline LFO nanostructures. In the sol–gel method, a precalcination process at 500 °C before calcination at 600 °C is capable of improving the crystallinity of nanoporous LFO powders; yet, it is difficult to form nanocrystalline LFO merely with a calcination process at 500 °C [26]. The coprecipitation method is normally used to prepare doped-LFO perovskite oxides with a requirement of high-temperature calcination. Even though it is hard to obtain pure-phase LFO without the use of complexant regents, the coprecipitation method is an ideal path to investigate the influence of experimental conditions and the mechanism of calcination.

In this work, we prepared crystalline LFO nanoparticles via a strictly controlled coprecipitation method with a calcination temperature of 500 °C. We investigated the effects of stoichiometric ratio, pH value, precipitants, complexant regent, and calcination temperature. We found that the precursors precipitated by aqueous NaOH or KOH at pH 10 successfully crystallized upon calcination at 500 °C, and the use of polyvinylpyrrolidone (PVP) had little influence on crystallization temperature. Moreover, an illusion of pure-phase crystalline LFO nanoparticles was observed in XRD patterns. We deduced that the structure of FeO_6_ octahedra formed in precursors is closely related to LFO’s crystallization temperature, which provides insight for the preparation of nanostructured perovskite oxides at low calcination temperatures.

## 2. Experimental Section

**Materials:** Crystalline LFO nanoparticles were prepared by a conventional coprecipitation method with calcination at different temperatures. In brief, as-received La(NO_3_)_3_·6H_2_O (99.9%, Macklin, Shanghai, China) and Fe(NO_3_)_3_·9H_2_O (99.9%, Macklin, Shanghai, China) with different La/Fe stoichiometric ratios were dissolved in Milli-Q water with stirring at 300 r·min^−1^ for 2 h using a magnetic stirrer. Aqueous alkali was then dropped into the obtained homogenized solution until pH 10 with stirring at 600 r·min^−1^ in an hour. The precipitates after centrifugation at 5000 rpm were thoroughly washed by Milli-Q water until pH 7 and subsequently dried at 60 °C for 24 h. Finally, the washed (pH 7) and unwashed (pH 10) precursors were both calcined at different temperatures for 6 h. NaOH (96%, Aladdin, Shanghai, China) and KOH (90%, Aladdin, Shanghai, China) were dissolved in Milli-Q water with the concentration of 6 M and then used to precipitate the precursors, and ammonia (25–28%, Aladdin, Shanghai, China) was used as a precipitant without dilution. Polyvinylpyrrolidone (PVP) with an average molecular weight of 40,000 (99%, ACMEC, Shanghai, China) was used as the complexant agent to regulate the processes of both precipitation and calcination.

**Characterization:** Morphologies of as-prepared LFO nanoparticles were investigated by a LEO 1530VP scanning electron microscope (SEM, Merlin Compact, Oberkochen, Germany) and Tecnai G2 F20 S-Twin high-resolution transmission electron microscope (HR-TEM, FEI, Hillsboro, OR, USA) with an energy-dispersive spectroscopy (EDS, Oberkochen, Germany) detector. The crystal structures of all samples were collected via a Bruker AXS D8 Advance X-ray diffractometer (XRD, Billerica, MA, USA) equipped with a Cu Kα radiation source (λ = 1.5406 Å), using a 2θ angle range from 20° to 80° in continuous scanning mode (step length 0.02) at scanning speeds of 0.11°/s and 0.012°/s, respectively. The Rietveld refinement was employed to study the lattice parameters using GSAS, and the Scherrer equation was adopted to evaluate the particle size. Raman spectra were collected by XploRA INV (HORIBA, Jobin Yvon, Palaiseau, France) with a laser wavelength of 473 nm. X-ray photoelectron spectroscopy (XPS, Shimadzu, Hongkong, China) analysis was performed by an Axis Supra with Al Kα radiation, and the binding energy was corrected using the C 1s level at 284.8 eV as an internal standard.

## 3. Results

### 3.1. Preparation of Crystalline LFO Nanoparticles

**Figure 1:**
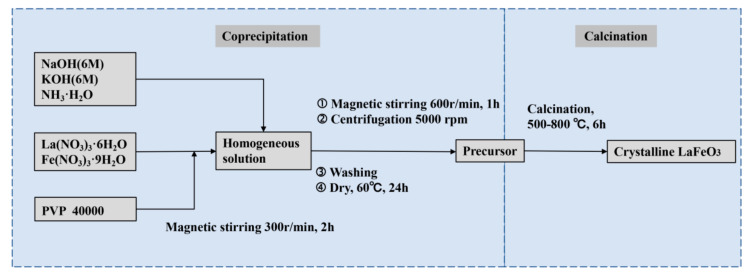
Scheme of the synthesis route.

As shown in the synthesis route in Figure 1, we prepared the precursors of crystalline LFO nanoparticles via a conventional coprecipitation method where aqueous sodium and potassium hydroxide, as well as ammonia, were used as precipitants, respectively. Subsequently, the as-prepared precursors were calcined at different temperatures to complete crystallization. Table 1 presents the phases of products identified by X-ray diffraction spectroscopy (XRD) under different conditions, including La/Fe stoichiometric ratios, pH values, and calcination temperatures, respectively. It unveils that the crystallized LFO phase was formed when the La/Fe stoichiometric ratio was higher than 3:7 and the calcination temperature was above 500 °C.

### 3.2. Influences of La/Fe Stoichiometric Ratios

**Figure 2:**
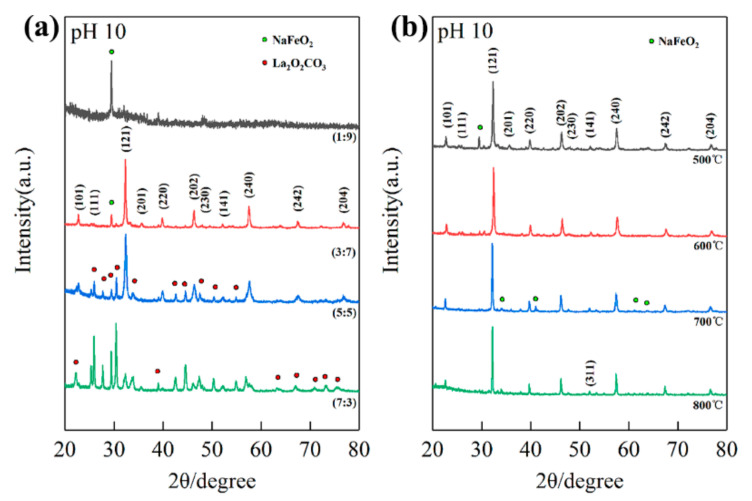
The XRD patterns of different La/Fe ratios after calcination at 500 °C (**a**) and the XRD patterns of La/Fe 3:7 after calcination at different temperatures (**b**).

Figure 2a shows the XRD patterns of different La/Fe stoichiometric ratios after precipitation at pH 10 and calcination at 500 °C. As the La/Fe ratio increased from 1:9 to 7:3, La-contained phases such as LaFeO_3_ and La_2_O_2_CO_3_ emerged gradually. The single diffraction peak at 29.6° in the XRD pattern of La/Fe 1:9 should be attributed to NaFeO_2_ according to JCPDS 13-0521, indicating that the La concentration is insufficient to form crystalline LaFeO_3_. The diffraction peaks at 22.6°, 25.3°, 32.2°, 36.1°, 39.7°, 46.1°, 47.6°, 51.9°, 57.4°, 67.3°, and 76.6° in the XRD pattern of La/Fe ratio 3:7 should be attributed to the crystal planes (101), (111), (121), (201), (220), (202), (230), (141), (240), (242), and (204) in orthogonal LaFeO_3_, respectively, in accordance with JCPDS 37-1493. It indicates the formation of crystalline LaFeO_3_. The other diffraction peaks at 29.5°should be attributed to the crystal plane (120) in NaFeO_2_, indicating that NaFeO_2_ was formed as well. It further implies that crystalline LaFeO_3_ and NaFeO_2_ were formed together during calcination at 500 °C. Except the characteristic diffraction peaks of LaFeO_3_ and NaFeO_2_, the presence of diffraction peaks at 22.2°, 25.1°, 27.6°, 30.3°, 33.6°, 42.5°, 44.4°, 47.4°, 50.2°, 52.1°, 54.7°, 56.9°, 57.8°, 63.2°, 66.9°, 70.8°, and 73.1° in the XRD pattern of La/Fe 7:3 should be attributed to the crystal planes (004), (100), (101), (102), (103), (006), (106), (110), (112), (107), (116), (201), (203), (1110), (206), (211), and (213) in hexagonal La_2_O_2_CO_3_, respectively, in accordance with JCPDS 37-0804. It indicates the formation of crystalline La_2_O_2_CO_3_ resulting from the excess La concentration. In addition, Figure S1b shows the XRD patterns of La/Fe 5:5 (pH = 7) after calcination at different temperatures. The characteristic diffraction peaks of La_2_O_2_CO_3_ first emerged after calcination at 500 °C, and then the characteristic diffraction peaks of LaFeO_3_ emerged after calcination at 600 °C. It indicates that La_2_O_2_CO_3_ and LaFeO_3_ crystallized during calcination at 500 °C and 600 °C, respectively. The single weak diffraction peak at about 40° in the XRD pattern of La/Fe 5:5 (pH = 7) after calcination at 500 °C is notable, which should be attributed to the crystal plane (220) in LaFeO_3_. It implies that the element of Fe might exist in amorphous LaFeO_3_.

### 3.3. Influence of pH Values and Calcination Temperatures

Next, we analyzed the XRD patterns of different pH values and different calcination temperatures, respectively. The characteristic diffraction peaks of LaFeO_3_ emerged in the XRD patterns of La/Fe 1:9 (Figure S1a) after calcination at 600 °C (pH 10) and 700 °C (pH 7), respectively, and emerged in the XRD patterns of both La/Fe 3:7 (Figure 2a) and 5:5 (Figure S1b) after calcination at 500 °C (pH 10) and 600 °C (pH 7), respectively. It strongly indicates that high pH values benefitted crystallization at lower calcination temperatures. Figure S1a shows the presence of Fe_2_O_3_ and NaFeO_2_ at pH 7 and pH 10, respectively, in the XRD pattern of La/Fe 1:9 after calcination at 700 °C, further indicating the influence of pH value. Figure S1b,c show that the characteristic diffraction peaks of La_2_O_2_CO_3_ vanished and the characteristic diffraction peaks of La_2_O_3_ emerged after calcination (La/Fe 5:5 and 7:3) above 700 °C, indicating the conversion from La_2_O_2_CO_3_ to La_2_O_3_. As shown in Figure 2b, the two characteristic diffraction peaks of NaFeO_2_ at 22.9° vanished when the calcination temperature increased from 500 °C to 600 °C, and another four characteristic diffraction peaks of NaFeO_2_ at 34.7°, 36.1°, 41.1°, and 61.2° emerged when the calcination temperature further increased to 700 °C. It indicates the significant influence of calcination temperature on NaFeO_2_ crystallization. Particularly, a single orthogonal perovskite LaFeO_3_ was observed in the XRD patterns of La/Fe 3:7 (pH 7) after calcination at 600 °C (Figure S2), which is prone to induce the illusion of purely crystalline LaFeO_3_. To determine lattice parameters of LFO, we further collected the XRD pattern of both La/Fe 3:7 pH 10 and 7 at a lower scanning speed (0.012°/s) via Rietveld refinement (Figure S3). Table S1 presents the lattice parameters of LFO in the sample of La/Fe 3:7 pH 10 after calcination at 500 °C (*Pnma, a* = 5.5536, *b* = 7.8490, *c* = 5.5503, *R_p_* = 5.72%, *R_wp_* = 7.87%) and in the sample of La/Fe 3:7 pH 7 after calcination at 600 °C (*Pbnm, a* = 5.5536, *b* = 5.5503, *c* = 7.8490, *R_p_* = 4.52%, *R_wp_* = 5.73%), where both of them are similar to those reported in the literature [5,27]. Furthermore, the average length of the Fe–O bond in the sample La/Fe 3:7 (pH 7) after calcination at 600 °C (2.084 Å) was larger than that in the sample La/Fe 3:7 (pH 10) after calcination at 500 °C (2.015 Å), whereas the unit cell volume of LFO in the sample La/Fe 3:7 (pH 7) after calcination at 600 °C (241.741 Å^3^) was less than that in the sample La/Fe 3:7 (pH 10) after calcination at 500 °C (241.015 Å^3^). Moreover, Table S3 shows that the crystallinity of the sample La/Fe 3:7 (pH 7) after calcination at 600 °C was merely 28.35%, significantly less than that of the sample La/Fe 3:7 (pH 10) after calcination at 500 °C (75.32%). This indicates that the quality of the obtained LaFeO_3_ perovskite in the sample La/Fe 3:7 (pH 7) after calcination at 600 °C was lower than that in the sample La/Fe 3:7 (pH 10) after calcination at 500 °C; it also indicates more defects in the sample La/Fe 3:7 (pH 7) after calcination at 600 °C in comparison with the sample La/Fe 3:7 (pH 10) after calcination at 500 °C.

**Figure 3:**
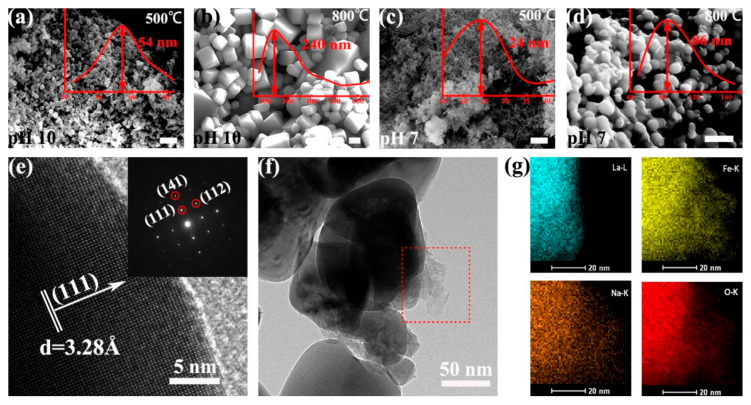
SEM images (**a**–**d**), HR-TEM and SAED images (**e**,**f**) of the LFO (La/Fe 3:7) calcined at 500 °C and 800 °C, respectively, and element mapping (**g**) of Fe, La, Na, and O on the surface of crystalline LFO nanoparticles after calcination at 800 °C. The inset red curves are the size distribution of the LFO nanoparticles. The scale bar is 300 nm in both SEM images.

We then investigated the morphologies of the as-prepared LFO (La/Fe 3:7) after calcination at different temperatures using scanning electron microscopy (SEM) and high-resolution transmission electron microscopy (HR-TEM). We observed that calcination temperature and pH value had remarkable influences on the shape of the LFO nanoparticles. As shown in Figure 3a–d, the shape of as-prepared LFO (pH 10) resembled uniform nanoparticles (average diameter ~54 nm) and cubic-like nanoparticles (average diameter ~240 nm) after calcination at 500 °C and 800 °C, respectively. In contrast, the shape of as-prepared LFO (pH 7) resembled ultrasmall nanoparticles (average diameter ~24 nm) and spherical nanoparticles (average diameter ~86 nm) after calcination at 500 °C and 800 °C, respectively. We further calculated the average particle size of LFO in the sample La/Fe 3:7 and pH 10 after calcination at 500 °C from the XRD pattern in Figure S3 using the Scherrer formula. As shown in Table S2, the average particle size of LFO was ~29 nm, slightly smaller than the statistical diameter (~54 nm) from the SEM image in Figure 3a. The HR-TEM image shows that the as-prepared LFO (pH 10) contained an amorphous phase and crystalline phase, and the selected-area electron diffraction (SAED) pattern confirmed that the crystalline phase was LFO (Figure 3e,f). Energy-dispersive spectroscopy (EDS) analysis further revealed the distribution of Fe, La, Na, and O at the interface between crystalline LFO and the amorphous phase (Figure 3g). It is worth noting that the La distribution constrained inside the crystalline LFO, whereas Fe, Na, and O elements distributed in both crystalline LFO and the amorphous phase. It indicates the amorphous NaFeO_2_ located outside the crystalline LFO nanoparticles and the formation of NaFeO_2_/LFO heterostructures. In addition, Table S4 lists the atomic contents of Fe, La, and O in the precursors of La/Fe 3:7 (pH 10 and 7) before and after calcination, respectively. The O-content in the precursor (pH 7) was 63.3% and then increased to 68.6% after calcination at 600 °C, whereas the O-content in the precursor (pH 10) remained at 61.4% even after calcination at 500 °C.

### 3.4. Influence of Precipitants

**Figure 4:**
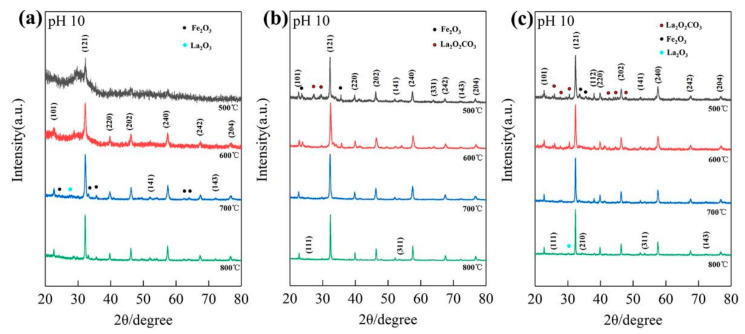
The XRD patterns of samples (La/Fe 3:7) precipitated by ammonia (**a**) and KOH solution (**b**), and NaOH solution with the use of PVP (**c**), after calcination at different temperatures.

To investigate the influence of precipitants, we further prepared precursors (La/Fe 3:7) using aqueous potassium hydroxide and ammonia. When precipitated by ammonia, a single diffraction peak at 32.1° of the LFO phase was observed after calcination at 500 °C, and a series of characteristic diffraction peaks assigned to perovskite LFO emerged after calcination at 600 °C (Figure 4a). As the calcination temperature increased to 700 °C, the characteristic diffraction peaks of the LFO phase significantly strengthened, coupling with the presence of the Fe_2_O_3_ phase and La_2_O_3_ phase. SEM images in Figure S4 demonstrate that the size of as-prepared LFO nanoparticles after calcination at 800 °C was considerably larger than that after calcination at 500 °C.

As opposed to the precipitated ammonia and NaOH solution, the phases of LFO, Fe_2_O_3_, and La_2_O_2_CO_3_ emerged when precipitated by KOH solution (6 M) after calcination at 500 °C. Figure 4b shows that the phase of perovskite LFO strengthened, whereas the phases of Fe_2_O_3_ and La_2_O_2_CO_3_ vanished after calcination at 700 °C. Figure S5 reveals that the size of as-prepared LFO nanoparticles after calcination 800 °C was around 100 nm, far smaller than that of LFO nanoparticles prepared by ammonia or NaOH solution. HR-TEM images in Figure S6 show that the as-prepared LFO after calcination at 800 °C comprised the crystallized phase and amorphous phase, similar to those of the LFO prepared by NaOH solution after calcination at 800 °C. In detail, the lattice planes with a distance of d~3.57 Å were in good agreement with the (111) plane of the perovskite LFO, which is clear evidence of crystalline LFO nanoparticles. Fe, La, K, and O mapping images demonstrate that the amorphous phase contained iron and potassium, similar to those of as-prepared LFO heterostructures after calcination at 800 °C.

### 3.5. Influence of Polyvinylpyrrolidone

We also prepared precursors (La/Fe 3:7) precipitated by aqueous NaOH (pH 10) with the use of polyvinylpyrrolidone (PVP) to investigate the influence of complexant agents. As shown in Figure 4c, the phases of Fe_2_O_3_ and La_2_O_2_CO_3_ emerged with the phase of perovskite LFO with the use of PVP after calcination at 500 °C, whereas the phase of NaFeO_2_ emerged with the phase of perovskite LFO without the use of PVP. The XRD patterns of the samples with the use of PVP presented a single orthogonal perovskite LaFeO_3_ with strengthened diffraction peaks after calcination above 700 °C. Figure S7 shows morphologies of the as-prepared LFO nanoparticles with the use of PVP after calcination at 500 °C and 800 °C, respectively. It shows that crystalline LFO nanoparticles were formed after calcination at 800 °C, similar to the effects of using aqueous KOH as a precipitant.

### 3.6. Mechanism Investigation

**Figure 5:**
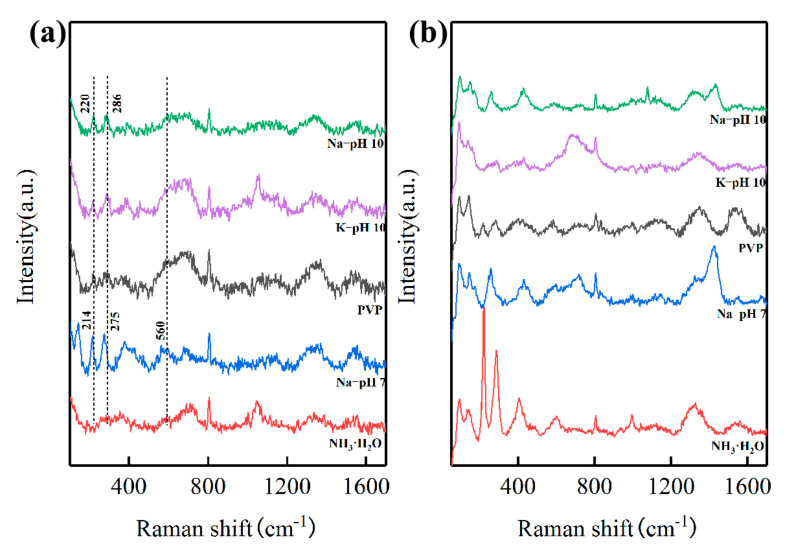
Raman spectra of the precursors before (**a**) and after (**b**) calcination at 800 °C, respetively.

In order to investigate the mechanism of lowering the calcination temperature, the precursors prepared under different conditions before and after calcination at 800 °C were probed using Raman spectroscopy, which is sensitive to the LFO-based perovskite structure [28,29,30]. The Raman spectra of the precursors prepared under different conditions after calcination at 800 °C are shown in Figure 5b. The Raman modes for orthorhombic LFO perovskite structures at 100~200 cm^−1^, 200~300 cm^−1^, 400~450 cm^−1^, 500~700 cm^−1^, and 1320~1350 cm^−1^ were observed in all samples, corresponding to La vibrations, oxygen octahedral tilt, oxygen octahedral bending vibrations, oxygen stretching vibrations, and the second-order scattering of oxygen vibrations, respectively.

Of note, Figure 5a shows the two important modes at 220 cm^−1^ and 286 cm^−1^ in the precursor prepared by NaOH solution (pH 10), indicating the formation of FeO_6_ octahedra. Identical peaks were observed in the precursor prepared by the KOH solution as well. However, these two peaks shifted to 214 cm^−1^ and 275 cm^−1^, respectively, when prepared by NaOH solution (pH 7), indicating that looser FeO_6_ octahedra were formed. Furthermore, another peak shift was also observed in the range from 500 cm^−1^ to 700 cm^−1^, indicating that the antisymmetric stretching motion of the oxygen octahedra in the precursor prepared at pH 10 was stronger than that in the precursor prepared at pH 7. The peak shifts in both ranges indicate that the atomic distance inside FeO_6_ octahedra in the precursor prepared at pH 10 was closer than that in the precursor prepared at pH 7, thus requiring less energy for the formation of perovskite LFO.

We further explored the X-ray photoelectron spectra (XPS) of as-prepared nanocrystalline LFO after calcination at 500 °C and the precursors prepared by NaOH solution at pH 10 and 7, respectively. As shown in Figure S8, two pairs of XPS peaks at ~855 eV (~851 eV) and ~838 eV (~835 eV) should be assigned to La 3d3/2 and 3d5/2, respectively. The XPS peaks at ~724 eV and ~711 eV should be assigned to Fe 2p1/2 and 2p3/2, respectively. In detail, the peaks of Fe 2p1/2 and Fe 2p3/2 in the precursor La/Fe 3:7 (pH 7) were 724.69 eV and 710.19 eV, respectively, slightly lower than those of 725.52 eV and 711.11 eV, respectively, in the precursor La/Fe 3:7 (pH 10). Considering that the values of Fe 2p1/2 and Fe 2p3/2 in the precursor La/Fe 3:7 (pH 10) is consistent with the values of Fe (III) in standard Fe_2_O_3_ [31], we speculate that the oxidation of Fe in precursor La/Fe 3:7 (pH 7) might be slightly less than 3+ Moreover, the peaks at 532.5 eV and 529.6 eV should be assigned to the absorbed O and lattice O, respectively, the characteristic peaks of O 1s in LFO [32,33]. In comparison with the precursor (pH 7), the shape of the O 1s spectrum in the precursor (pH 10) was closer to that in as-prepared LFO after calcination at 500 °C, indicating that the Fe–O structure in precursor (pH 10) was closer to that in LFO.

## 4. Discussion

Considering that coprecipitation is a facile and simple method for facilitating the mechanism investigation, we fabricated crystalline LFO nanoparticles using coprecipitation and calcination. Besides the calcination temperature, we investigated the major influence factors involving molar ratios of La and Fe, precipitators, and pH values of precursors in the process of coprecipitation. As shown in Table 1, the crystalline LFO emerged after calcination at 500 °C when the stoichiometric ratio of La:Fe was 3:7. In order to minimize the influence from unknown compounds that might be formed in the process of precipitation, we focused on the effects of other conditions when the stoichiometric ratio of La:Fe was 3:7. In particular, pH values of precursors had a significant effect on the sintering temperature in the ensuing process of calcination. The precursor precipitated at pH 10 was able to form crystalline LFO after calcination at 500 °C, whereas the precursor washed thoroughly to pH 7 remained amorphous under the same calcination conditions. The Raman study demonstrated that analogous FeO_6_ octahedra, a characteristic structure in perovskite LFO, were formed in both precursors prepared at pH 10 and 7. However, the observed blue shifts in the peaks of FeO_6_ octahedra in the precursor prepared at pH 10 were more compact than those in the precursor prepared at pH 7, indicating that the energy requirement of the precursor prepared at pH 10 to form crystalline LFO was less than that of the precursor prepared at pH 7. XPS spectra of the precursor prepared at pH 10, especially the O 1s spectrum, were more prone to those of LFO, further indicating the lower energy requirement for the formation of crystalline LFO. Therefore, the precursor prepared at pH 10 converted to crystalline LFO at a calcination temperature of 500 °C, lower than that of the precursor prepared at pH 10 (600 °C).

Lowering the calcination temperature is an avenue to effectively resist the growth and agglomeration of LFO nanostructures during the high-temperature process. Furthermore, lowering the calcination temperature would decrease the energy consumption and reduce carbon dioxide emissions as well. It should be noted that the presence of amorphous oxides was unable to be determined using XRD spectroscopy, which should be further confirmed by other auxiliary approaches (e.g., TEM and SEM). We prepared crystalline LFO nanoparticles with the stoichiometric ratio of La:Fe 3:7, and inevitably redundant iron compounds were formed as a corollary of the excessive Fe element (e.g., NaFeO_2_). Importantly, these redundant iron compounds were amorphous and hard to be detected by XRD spectroscopy, so an illusion of purely crystalline LaFeO_3_ was shown in XRD patterns with a variety of different experimental conditions. Based on the X-ray crystal structure analysis, we also observed that the calcination temperatures for the crystallization of La_2_O_2_CO_3_, LaFeO_3_, NaFeO_2_, and La_2_O_3_ were 400 °C, 500 °C, 500 °C, and 700 °C, respectively. Experimental conditions such as stoichiometric ratios, pH values, precipitants, and PVP would significantly influence the crystallization temperature, which could be utilized for crystallization in certain sequences.

## 5. Conclusions

We prepared crystalline LaFeO_3_ nanoparticles via coprecipitation and calcination at 500 °C. Through investigating the experimental conditions, including stoichiometric ratio, pH value, precipitants, complexant regents, and the calcination temperature, we concluded the following: (1) crystalline LaFeO_3_ nanoparticles could be formed after calcination at 500 °C when the stoichiometric ratio La/Fe was higher than 3:7, coupling with the formation of crystalline NaFeO_2_; (2) coprecipitation at a high pH value was conducive to the crystallization of LaFeO_3_ at low calcination temperatures; (3) crystalline LaFeO_3_ nanoparticles could be formed after calcination at 500 °C when using either NaOH or KOH solutions as a precipitant at pH 10; (4) the use of PVP could influence the morphology of crystalline LaFeO_3_ nanoparticles instead of calcination temperature. We inferred from Raman spectra that the structure of FeO_6_ octahedra formed in the process of precipitation was associated with the calcination temperature. We also observed an illusion of pure-phase LaFeO_3_, where LFO-based heterostructures involved a crystalline phase of LaFeO_3_ and an amorphous phase of NaFeO_2_ or KFeO_2_, respectively. We anticipate that these results could improve the preparation of nanostructured perovskite oxides at low calcination temperatures.

## Figures and Tables

**Table 1:** Phases of products prepared by NaOH precipitation; NFO and LCO represent NaFeO_2_ and La_2_O_2_CO_3_, respectively.

La:Fe	pH	500 °C	600 °C	700 °C	800 °C
1:9	7			LFO/Fe_2_O_3_	LFO/Fe_2_O_3_
	10	NFO	LFO/NFO	LFO/NFO	LFO/NFO
3:7	7		LFO	LFO/NFO	LFO/NFO
	10	LFO/NFO	LFO/NFO	LFO/NFO	LFO/NFO
5:5	7	LCO	LFO/LCO	LFO/La_2_O_3_	LFO/La_2_O_3_
	10	LFO/LCO	LFO/LCO	LFO/La_2_O_3_	LFO/La_2_O_3_
7:3	7	LFO/LCO	LFO/LCO	LFO/La_2_O_3_	LFO/La_2_O_3_
	10	LFO/LCO	LFO/LCO	LFO/La_2_O_3_	LFO/La_2_O_3_
3:7/PVP	10	LFO/Fe_2_O_3_/LCO	LFO/Fe_2_O_3_/LCO	LFO/Fe_2_O_3_/LCO	LFO/Fe_2_O_3_/La_2_O_3_

## Data Availability

Not applicable.

## Supplementary Materials

The following are available online at https://www.mdpi.com/article/10.3390/ma14195534/s1, Figure S1: The XRD patterns of samples La/Fe 1:9 (a), 5:5 (b), and 7:3 (c) prepared at different pH values and different calcination temperatures; Figure S2: The XRD patterns of sample La/Fe 3:7 at pH 7 and different calcination temperatures; Figure S3: Combined XRD pattern and Rietveld refinement of the sample La/Fe 3:7 pH 10 after calcination at 500 °C (a) and pH 7 after calcination at 600 °C (b); Figure S4: SEM images of as-prepared LFO nanoparticles prepared by ammonia, after calcination at 500 °C (a) and 800 °C (b); Figure S5: SEM images of as-prepared LFO nanoparticles prepared by KOH solution, after calcination at 500 °C (a) and 800 °C (b); Figure S6: HR-TEM images (a, b) of the LFO (La/Fe 3:7) calcined at 800 °C, and element mapping of La, Fe, K, and O on the surface of crystalline LFO nanoparticles after calcination at 800 °C; Figure S7: SEM images of as-prepared LFO nanoparticles prepared by NaOH solution with the use of PVP, after calcination at 500 °C (a) and 800 °C (b); Figure S8: The XPS spectra of La, Fe, and O in the as-prepared LFO after calcination at 500 °C and the precursors prepared by NaOH solution at pH 10 and 7; Table S1: Rietveld refinement results of LFO in the sample La/Fe 3:7 pH 10 after calcination at 500 °C and pH 7 after calcination at 600 °C; Table S2: Calculated particle size of LFO in the sample La/Fe 3:7 pH 10 after calcination at 500 °C; Table S3: The crystallinity of the sample La/Fe 3:7 pH 7 after calcination at 600 °C and pH 10 after calcination at 500 °C; Table S4: The atomic contents of O, Fe, and La in the precursors of La/Fe 3:7 pH 10 and 7, before and after calcination at 600 °C and 500 °C.
