# Supplementary material for: Preparation of Crystalline LaFeO3 Nanoparticles at Low Calcination Temperature: Precursor and Synthesis Parameter Effects
Source: Materials (Basel). 2021 Sep 24;14(19):5534. doi: 10.3390/ma14195534 (PMC8509417; doi:10.3390/ma14195534)
Supplement: Supplementary file 1 [file materials-14-05534-s001.zip › materials-1387263 supplementary.pdf]

Supplementary materials

Preparation of crystalline $LaFeO_3$ nanoparticles at low calcination temperature: precursor and synthesis parameter effects

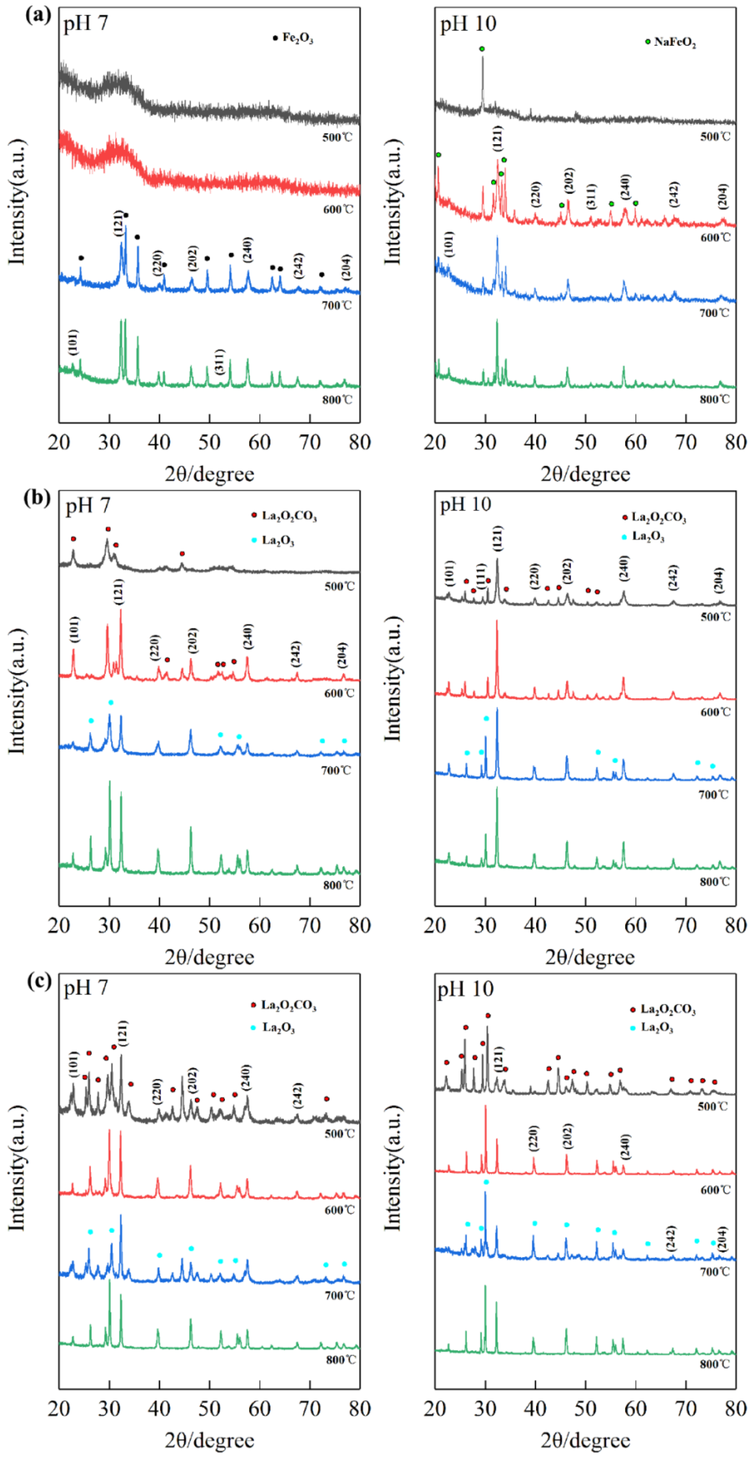

Figure S1. The XRD patterns of samples La/Fe 1:9 (a), 5:5 (b), 7:3 (c) prepared at different pH values and different calcination temperatures.

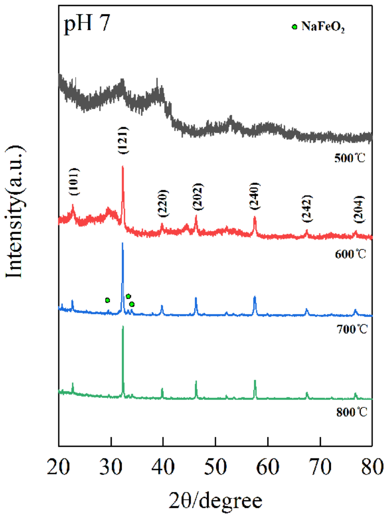

Figure S2. The XRD patterns of samples La/Fe 3:7 at pH 7 and different calcination temperatures.

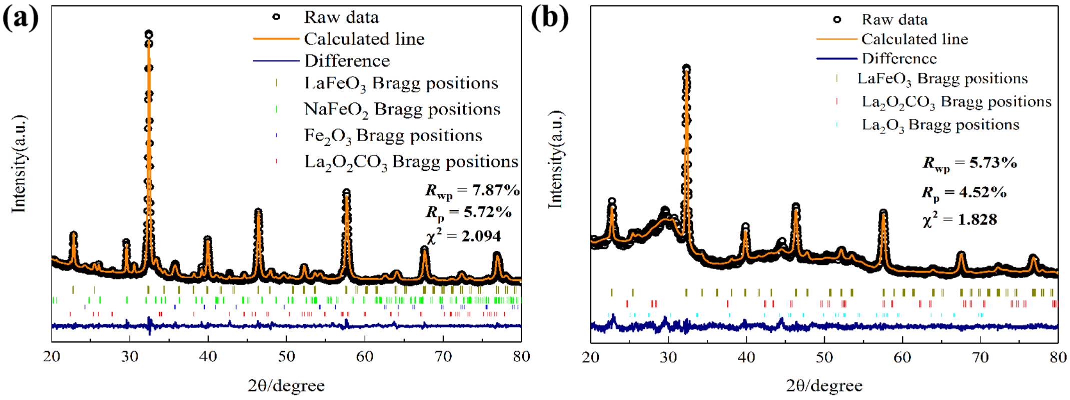

Figure S3. Combined XRD pattern and Rietveld refinement of the sample La/Fe 3:7 pH 10 after calcination at 500 °C (a) and pH 7 after calcination at 600 °C (b), respectively.

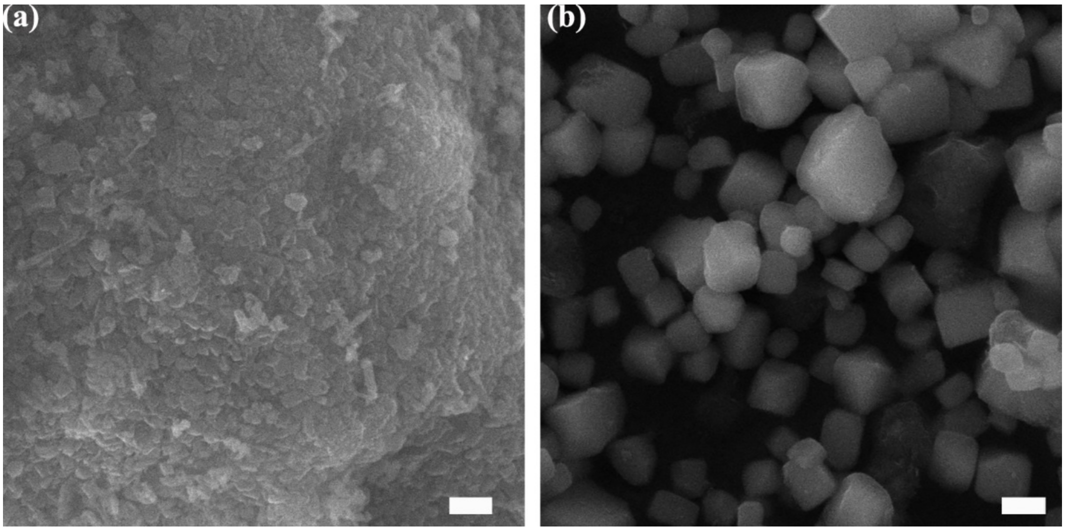

Figure S4. SEM images of as-prepared LFO nanoparticles prepared by ammonia, after calcination at 500 °C (a) and 800 °C (b), respectively. The scale bar is 300 nm in both SEM images.

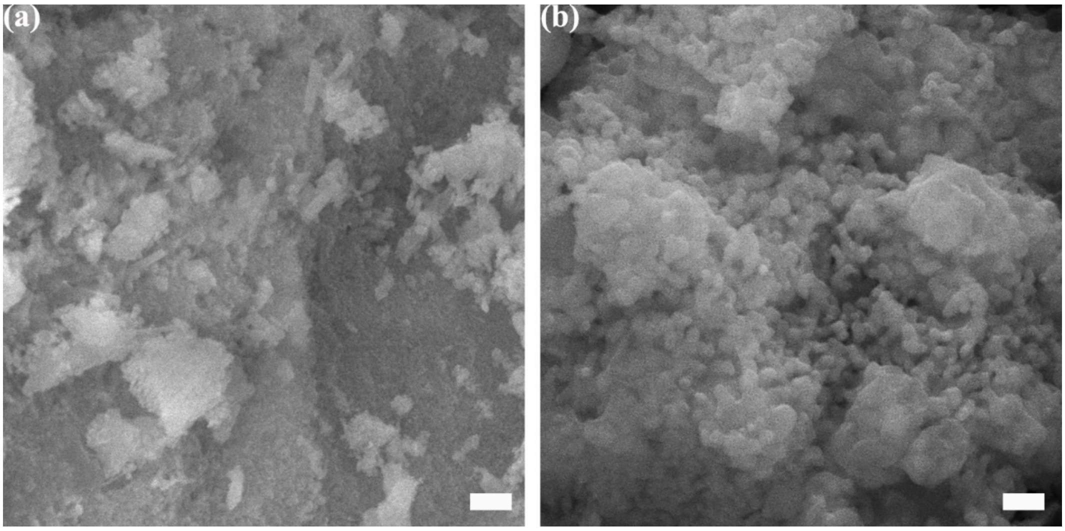

Figure S5. SEM images of as-prepared LFO nanoparticles prepared by KOH solution, after calcined at 500 °C (a) and 800 °C (b), respectively. The scale bar is 300 nm in both SEM images.

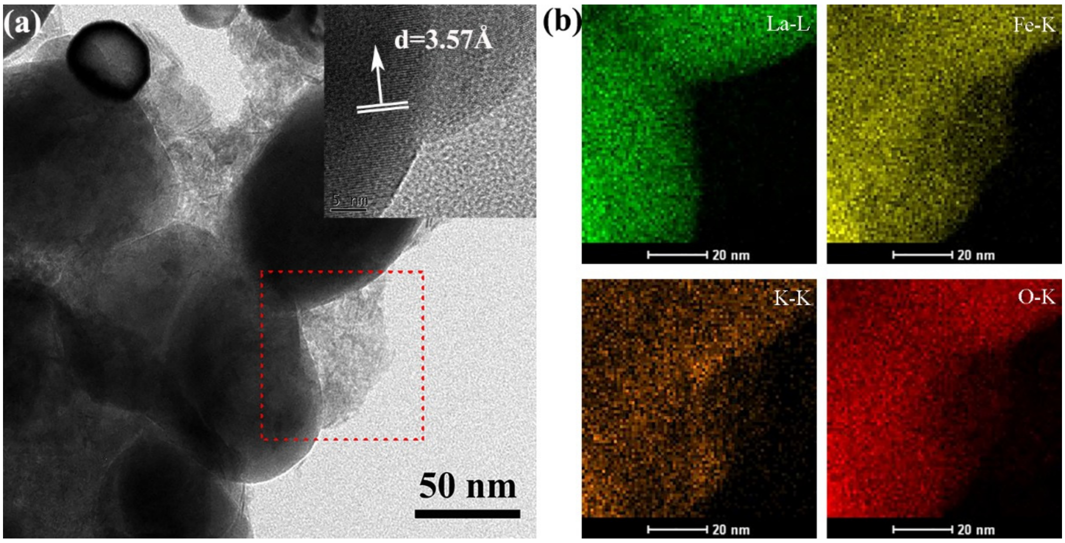

Figure S6. HR-TEM images (a, b) of the LFO (La/Fe 3:7) calcined at 800 °C, and element mapping of La, Fe, K, O on the surface of crystalline LFO nanoparticles after calcination at 800 °C.

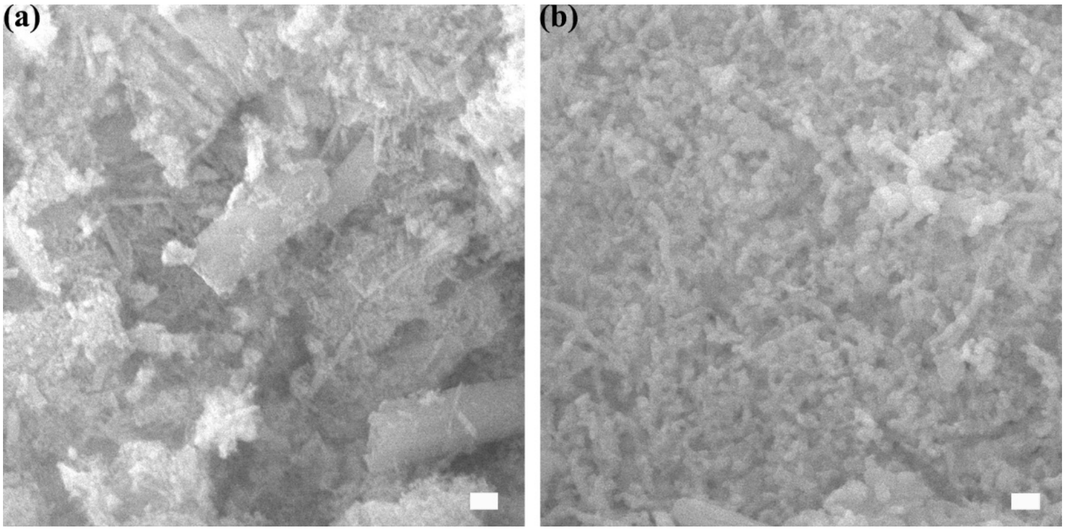

Figure S7. SEM images of as-prepared LFO nanoparticles prepared by NaOH solution with the use of PVP, after calcination at 500 °C (a) and 800 °C (b), respectively. The scale bar is 200 nm in both SEM images.

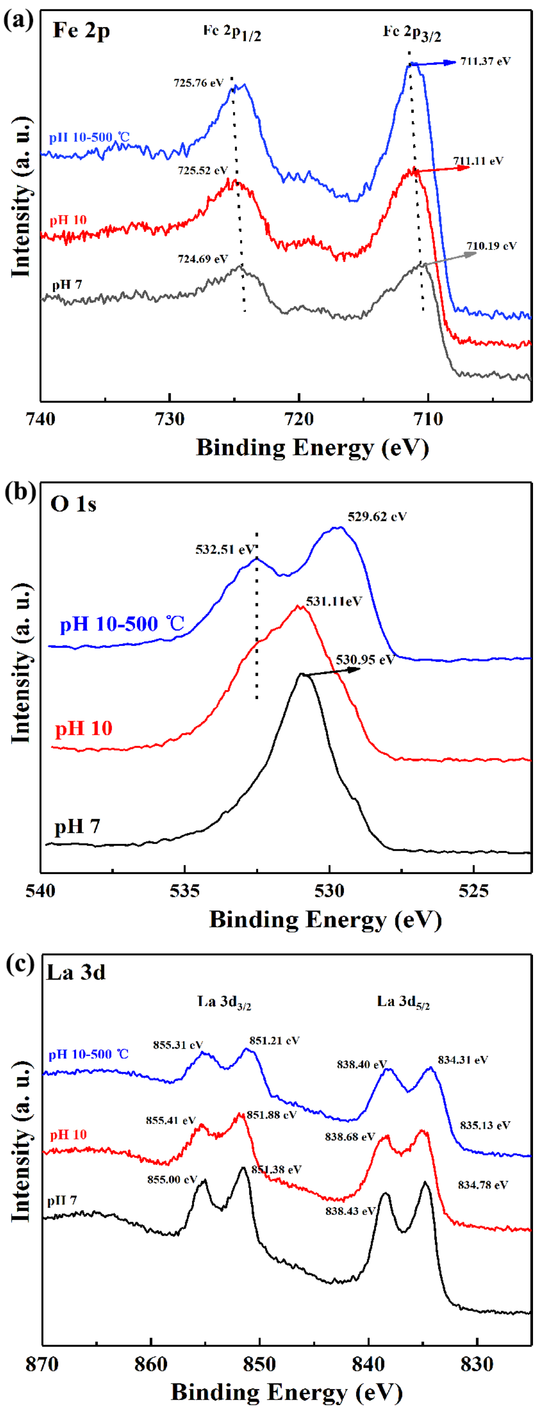

Figure S8. The XPS spectra of La, Fe, O in the as-prepared LFO after calcination at 500 °C and the precursors prepared by NaOH solution at pH 10 and 7, respectively.

Table S1. Rietveld refinement results of LFO in the sample La/Fe 3:7 pH 10 after calcination at 500 °C and pH 7 after calcination at 600 °C, respectively.

Compound		$LaFeO_3$ pH 10, 500 °C	$NaFeO_2$ pH 10, 500 °C	$LaFeO_3$ pH 7, 600 °C
Space Group		Pnma	Pna (21)	Pbnm
Lattice parameters (Å)	a	5.5536±0.0004	5.6004±0.0011	5.5536±0.0002
	b	7.8490±0.0007	7.1548±0.0007	5.5503±0.0004
	c	5.5503±0.0004	5.3804±0.0008	7.8490±0.0011
Unit cell volume, V ($Å^3$)		241.015	217.302	241.741
Bond length (Fe-O)		1.9631		1.88
		1.9631		2.397
		2.184		1.88
		1.895		2.397
		2.184		1.975
		1.895		1.975
Average bond length (Å)		2.015		2.084
R factors (%)	R_p	5.72		4.52
	R_{wp}	7.87		5.73
	χ^2	2.094		1.828

Table S2. Calculated particle size of LFO in the sample La/Fe 3:7 pH 10 after calcination at 500 °C.

Scherrer formula $D = \frac{K\gamma}{B \cos\theta}$			
B	0.247	0.335	0.371
θ	16.15	23.16	28.77
K	0.943		
γ		1.54056 Å	
Calculated particle size	35.08 nm	27.02 nm	25.59 nm
Average particle size		29.23 nm	

Where B is the peak FWHM, θ is the degree, K is the Scherrer constant for cubic particles, and γ is wavelength.

Table S3. The crystallinity of the sample La/Fe 3:7 pH 7 after calcination at 600 ºC and pH 10 after calcination at 500 ºC, respectively.

$$Crystallinity = \frac{Area\ of\ crystalline\ peaks}{Area\ of\ all\ peaks(crystalline + Amorphous)}\ x100\%$$

	pH 10, 500°C	pH 7, 600°C
$Area\ of\ crystalline\ peaks$	14921.886	9382.393
$Area\ of\ all\ peaks$	19811.032	33094.863
$Crystallinity$ (%)	75.32	28.35

Table S4. The atomic contents of O, Fe, La in the precursors of La/Fe 3:7 pH 10 and 7, respectively, before and after calcination at 600 °C and 500 °C, respectively.

	precursor, pH 7	precursor pH, 10	pH 7, calcination at 600 °C	pH 10, calcination at 500 °C
O	63.3	61.4	68.6	61.4
Fe	26.7	24.8	21.7	25.3
La	10	13.8	9.7	13.3
